# Characterization of a Novel Approach for Neonatal Hematocrit Screening Based on Penetration Velocity in Lateral Flow Test Strip

**DOI:** 10.3390/s23052813

**Published:** 2023-03-04

**Authors:** Lorenzo Zucchini, Miloš Ajčević, Carlos Daniel Coda Zabetta, Chiara Greco, Cristina Fernetti, Carlo Moretto, Simone Pennini, Agostino Accardo

**Affiliations:** 1 Department of Engineering and Architecture, University of Trieste, 34127 Trieste, Italy; 2 Bilimetrix s.r.l., 34149 Trieste, Italy; 3 Prodigys Technology s.r.l., 34149 Trieste, Italy

**Keywords:** hematocrit, lateral flow, neonatal care, point-of-care, LMICs

## Abstract

Hematocrit (HCT) is a crucial parameter for both adult and pediatric patients, indicating potentially severe pathological conditions. Most common methods for HCT assessment are microhematocrit and automated analyzers; however, developing countries present specific needs often not addressed by these technologies. Paper-based devices can be suitable for those environments being inexpensive, rapid, easy to use, and portable. The aim of this study is to describe and validate against a reference method, a novel HCT estimation method based on penetration velocity in lateral flow test strips complying with the requirements in low- or middle-income country (LMIC) scenarios. To calibrate and test the proposed method, 145 blood samples of 105 healthy neonates with gestational age greater than 37 weeks were collected (29 calibration set, 116 test set) in the range of HCT values (31.6–72.5%). The time difference (Δt) from the whole blood sample loading into the test strip instant till the nitrocellulose membrane saturation instant was measured by a reflectance meter. A nonlinear relation was observed between HCT and Δt and was estimated by a third-degree polynomial equation (R^2^ = 0.91) valid in 30% to 70% HCT interval. The proposed model was subsequently used to estimate HCT values on the test set showing a good agreement between the estimated HCT and the HCT measured by the reference method (r = 0.87, *p* < 0.001), with a low mean difference of 0.53 ± 5.04% and a slight trend of overestimation for higher hematocrit values. The mean absolute error was 4.29%, while the maximum absolute error was 10.69%. Although the proposed method did not present a sufficient accuracy to be used for diagnostic purposes, it could be suitable as a fast, low-cost, easy-to-use screening tool especially in LMIC scenarios.

## 1. Introduction

Hematocrit (HCT) is commonly used as a general indicator of a patient’s health status in relation to specific reference ranges for adults and newborns [1,2,3,4]. Deviations from these ranges can be used to aid diagnosing certain conditions related to health status in general clinical practice as well as in neonatology.

The main reasons for increased levels of HCT include dehydration, burns, diarrhea, postpartum eclampsia, and polycythemia vera; a high HCT level also indicates risk for heart and cerebral infarction because of hemoconcentration. Furthermore, increased HCT is a factor of thrombus formation and increases risk for thrombosis. This condition is especially dangerous for patients with artificial heart, in dialysis, and during open-heart surgery [4]. On the other hand, when the HCT level is reduced, symptoms of anemia and bleeding are usually suspected, as well as diseases of the bone marrow, leukemia, malnutrition, and overhydration. Furthermore, each change of HCT value affects the safe control of the blood pump [4,5,6].

This physical parameter is commonly used, for example, to decide whether to administer blood transfusion or partial exchange transfusion [7], and it should be considered in the presence of risk factors of numerous pathological conditions, such as hyperbilirubinemia [8,9].

Since changes in HCT reflect acute or chronic alterations in the patient’s state of health, a quick HCT estimation is critical to establish a prompt and adequate approach [7] or, at least, to suggest further investigations. Therefore, hematocrit could have even more importance and significance in neonatology, although recent literature shows that it may not yet be used to its full potential; for example, the HCT of infants under phototherapy is often not thoroughly considered in clinical practice: Lamola et al. [8] speculate that going from a HCT of 40% to 60% could mean a twofold reduction in the absorption of light by bilirubin; in other words, a twofold increase in irradiance or duration of exposure would be required to achieve the same amount of light absorbed by bilirubin for an infant with an HCT of 60% versus an infant with an HCT of 40%.

Nowadays, there are two most common approaches for HCT measurement in clinical use: (I) direct measurement by centrifugation of a blood-filled microcapillary tube and visual examination using a ruler (micro-HCT) and (II) automated calculation performed by modern blood analyzers [7,10,11]. Both are considered accurate and reliable enough to be used in clinical decision processes, although presenting some disadvantages: the first (I), being completely manual, requires the presence of a skilled operator; hence, it is susceptible to operator-related errors or technical errors, such as incorrect positioning of the capillary in rotors or incorrect setting of the centrifuge. Two other non-negligible downsides are biohazard risk introduced by broken glass capillaries and errors due to aberrant red blood cell morphologies [7,12]. The second (II) is susceptible to overestimation if certain clinical conditions are present, such as high concentration of platelets or white blood cells; on the other hand, this method is susceptible to underestimation in the presence of red blood cell agglutinates [7,13]. Another concern is with regard to consistency and agreement between different methods. In particular, despite being widely used, point-of-care automated blood analyzers based on different analytical methods can produce divergent results, which can lead to different clinical decisions [3,14].

Other than these weaknesses, the most interesting aspects for this study are turnaround time and complexity: for micro-HCT, the minimum technical time to execute the measure could be about 10 min, having a dedicated operator that collects the blood sample, fills and seals the capillary tube, centrifuges it, and reads the resulting centrifuged sample without any downtime. However, the time taken to complete this process is easily two- or threefold increased due to other parallel activities, and depending on the organizational aspects of the facility, the interval between collection and reading influences results itself, as blood changes its properties over time [12]. Conversely, for automated methods, turnaround time is strongly dependent on the sample collecting and handling process, laboratory distance, and quality of the instrumentation available; in this case, complexity is obviously much greater.

These aspects could considerably limit the applicability of the aforementioned methods in low- and middle-income countries (LMICs), which often lack instrumentation, facilities, and trained professionals and where healthcare access is generally poor [15,16,17]. Few alternative technologies have been developed to address the restrictions and constraints specific to LMICs, among them paper-based devices; in particular, lateral flow assays are rising in scientific and clinical interest. Recent applications of paper-based devices were found in the literature, capable of detecting proteins [18], microRNA biomarkers [19], micronutrients in food [20], and HIV virions [21].

Lateral flow test strips have many advantages: they require very small samples, the time necessary to obtain a result is shorter than the traditional laboratory turnaround time, they do not require or require very little consumables or reagents, they are easier to use and maintain than laboratory equipment, and they are inexpensive and easier to dispose of; these design features become constraints to be considered in LMICs, as evidenced in the IEEE 21st International Conference on Nanotechnology [22] and stated by Murray and Mace [23]. An aspect of interest for this study is that, in most cases, lateral flow assays provide qualitative or semiquantitative outputs, for which human intervention is necessary to adequately interpret the results. For this reason, a method based on lateral flow test strips that extends their advantages by integrating automation and quantitative response capabilities is desirable in LMICs.

Four previous studies were found in the literature: Berry S.B. et al. [24] developed a low-cost thermometer-style device that enables semiquantitative evaluation of HCT in 30 min from a 50 μL sample, working with HCTs in the 28–57% range; further refinement [25] improved device features, reducing both the sample size and the time required to 10 μL and 10 min, respectively. Differently, Del Ben F. et al. [26] proposed a method based on image analysis requiring a scanner and filter paper for dried blood spot collection, which resulted in good accuracy, precision, and reproducibility within 23–48% HCT interval. Then, Miller IV, J.H. [27] used Whatman 903 filter paper and a scientific-grade spectrometer to calculate hematocrit from dried blood spots in the 25–75% range, taking a 50 µL whole blood sample. Finally, Punter-Villagrasa J. et al. [28] presented a solution for the instantaneous detection of HCT based on three electrodes measuring the impedance of a 50 µL whole blood sample, capable of giving a −0.96 Pearson’s correlation coefficient testing a 14.2–50.6% input range, even though the results provided were limited to 45%.

The solutions reported in the aforementioned studies might also be valid for neonatal population, but they were principally focused on normal adult population ranges, which are typically lower than neonates [29]. Indeed, a large study [1] on more than 20,000 neonatal patients indicates a HCT reference range of 42–65%.

In general, assaying neonate HCT adds further constraints to the development of a valid alternative for, and not only, LMICs: (I) sample collection from neonates must be minimized in terms of both frequency and volume to reduce pain, stress induction, and infection probability; (II) neonates present higher HCTs than adults; (III) having a low-cost, low-complexity, and fast estimation method becomes more important as local health accessibility decreases.

The aim of this study is to describe and validate a novel HCT screening method based on penetration velocity in lateral flow test strips and quantified using a light reflectance meter.

## 2. Materials and Methods

### 2.1. HCT Estimation Method

**Figure 1:**
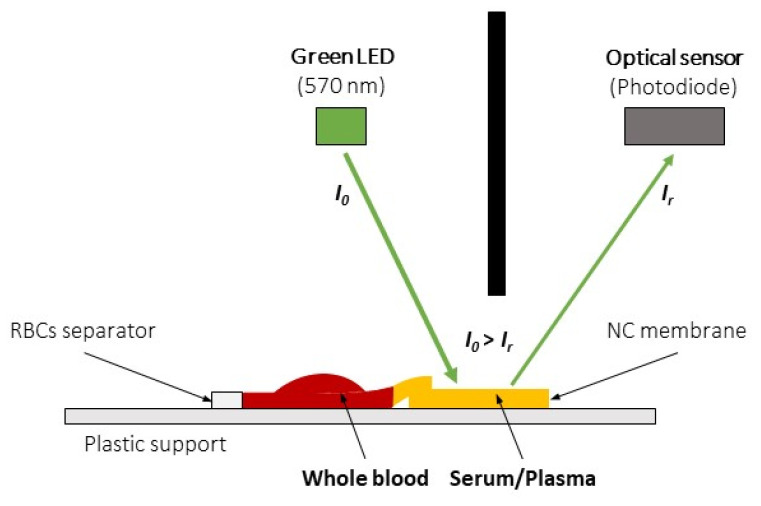
The optical measurement system. The LED light source irradiates the membrane filled with plasma forming the sample; an optical sensor positioned in the same chamber measures the amount of reflected light, enabling the calculus of sample absorbance.

**Figure 2:**
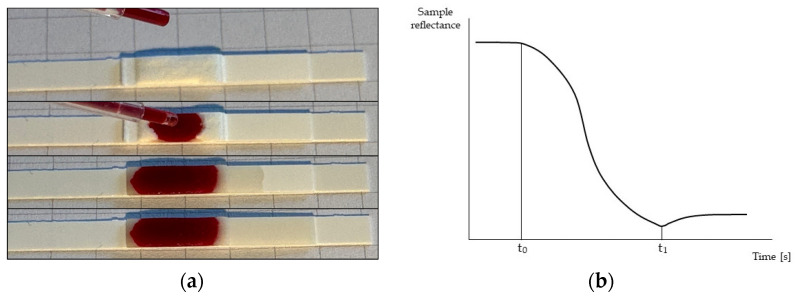
(**a**): from top to bottom, a blank strip having the filter in the center, in contact with the NC membrane extending to the right; the loading instant; plasma entering the membrane; the membrane has been saturated by plasma (**b**): characteristic reflectance-time pattern; t_0_ is defined as the instant in which plasma starts filling the NC membrane, associated with a decrease in reflectance; t_1_ is defined as the instant in which plasma saturates the NC membrane, associated with a minimum point in reflectance signal. Δt = t_1_ − t_0_.

The method for HCT determination was based on the plasma penetration velocity in a lateral flow test strip and a light reflectance meter. The lateral flow test strip was composed of a filter coupled with a membrane (Advanced Microdevices Pvt., Ambala, India). The filter was made of white fiberglass having a thickness of 570 ± 80 µm and a tare weight of 10.5 ± 3 mg/cm^3^; the membrane was made of nitrocellulose having a thickness of 105 ± 15 µm, a pore size of 15 µm, and a mean wicking time of 100 ± 25 s measured on a 4 cm distance with normal saline solution. The filter and the membrane were hand-cut to the same width and vertically coupled with a small overlap, allowing plasma to permeate from the filter to the membrane, as shown in Figure 1. The filter-membrane system was placed on a plastic support, guaranteeing mechanical rigidity during the experiment. A whole blood sample (35 µL) was loaded onto the filter, which retained the corpuscular part of the blood, allowing only the plasma to enter the NC membrane until its complete saturation, as illustrated in Figure 2a.

The entrance of plasma in the NC membrane was monitored using a light reflectance meter. The instrument irradiated the NC membrane with an LED emitting light at 570 nm; the reflected light intensity was measured by an optical sensor. A representation of the measuring principle is reported in Figure 1. The data for the present study were collected by instructing the optical system to perform four readings per second, therefore with a period of 250 ms. This sampling period was determined after a pilot analysis, which resulted in high measurement error using a sampling period of 500 ms. During the data collection, the trend of reflectance values was monitored, and the train of readings was interrupted when a series of equal readings occurred, a condition that indicated a stationary condition in the system, hence the saturation of the strip. Penetration velocity was indirectly calculated based on the time difference (Δt) between instant t_0_ and instant t_1_. The first indicated the entry of plasma into the NC membrane, which produced a progressive reduction of reflectance from the initial steady-state condition of the system. The latter indicated the complete saturation of the NC membrane: this final condition was identifiable as a minimum point in reflectance data. The nature of this behavior is physical and attributable to plasma reflectance: approaching membrane saturation, the reflectance was minimum as the quantity of plasma absorbing light was maximum, while immediately after saturation, a layer of plasma progressively formed over the membrane, producing a more reflective “glossy” surface. This temporary increase in reflectance anticipated the final equilibrium condition of the system and thus the end of a run. For this reason, all data points acquired after the minimum point t_1_ were irrelevant for our aim. In Figure 2b, a typical pattern of reflectance signal versus time is shown, and t_0_ and t_1_ are indicated.

If, during the analysis of the results, a run was particularly different due to lack of saturation of the strip or lack of blood flow through the filter, the run was rejected. In any case, the strips were observed at the end of each test to check the state of membrane saturation and any presence of hemolysis.

### 2.2. Data Acquisition, Calibration, and Clinical Test

**Figure 3:**
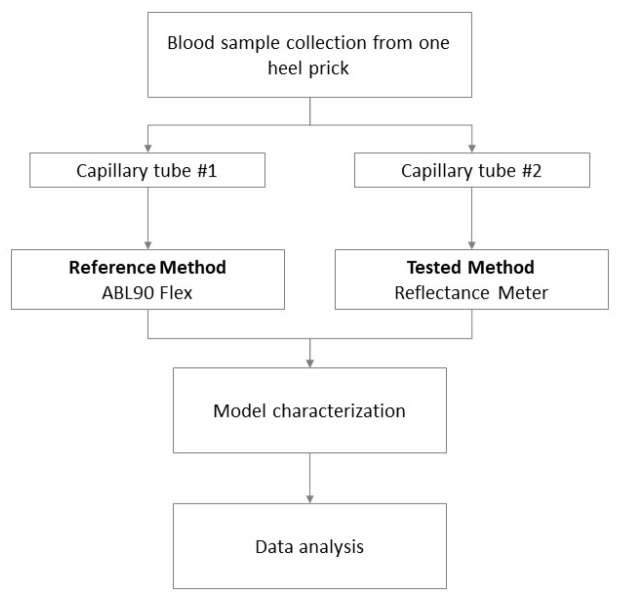
Workflow of procedure for data acquisition, model characterization, and clinical test.

Whole blood samples were collected from 105 healthy neonates over 37 weeks’ gestational age born at IRCCS Materno Infantile “Burlo Garofolo” of Trieste, Italy. Tests were performed in duplicate. However, knowing that each prick causes suffering and stress to the neonate, the number of samples collected during the study time was minimized by waiting for a blood sample to be available rather than collecting one strictly for the study purpose. The sample collection period was over 3 months, from February to May 2022. Blood samples were collected by puncture on the patient’s heel in two heparinized capillary tubes. One capillary was subjected to analysis with a blood gas analyzer (Radiometer ABL90 Flex), as a reference method for this study, while the other was estimated by the proposed method. In this case, the sample was transferred from the capillary tube to the test strip using an automatic pipette to minimize hemolysis and the formation of bubbles. Each measurement, with both one method and the other, was performed by trained personnel, and the data obtained were merged for comparison. With reference to the tested method, during the deposition of the sample, the personnel were instructed to observe the behavior of the blood drop permeating through the filter to detect any abnormality. Subsequently, at the end of each measurement performed by the reader, the strip was observed to detect incomplete saturation, hemolysis, or anomalies. Figure 3 summarizes the procedure from sample collection to data analysis.

Considering the total amount of acquired samples, 20% of them were used to perform the system calibration and calculate the model (calibration data), and the remaining 80% were used to perform a comparison with the reference method (test data). The samples used for the calibration were selected as described: the input HCT range was equally divided into four levels, from each of which at least five samples were randomly selected.

## 3. Results

A total of 192 runs were performed. Following the described protocol, a total of 28 runs were excluded due to visible hemolysis and erythrocyte presence in the plasma-filled membrane, while another 12 runs were excluded due to bubble formation during the deposition of the sample on the test strip. During data analysis, 7 more runs were excluded, showing outlying behavior. From the remaining 145 runs, as described in Materials and Methods, 29 were extracted randomly to create the model as 20% of the total, while 116 were used as test data.

**Figure 4:**
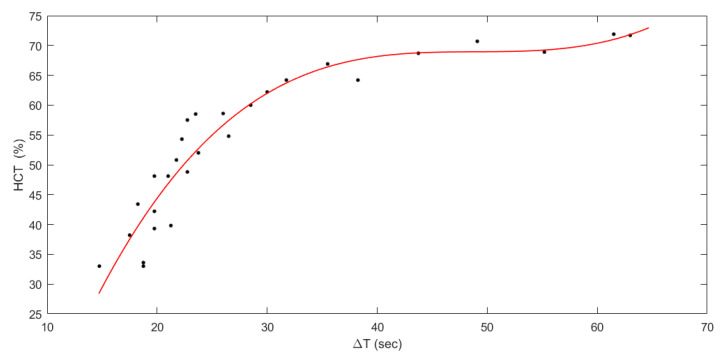
The measured HCT was plotted against Δt (black dots—calibration data) and fitted with a third-degree polynomial equation (red line).

In Figure 4, the measured HCT was plotted against Δt. A nonlinear relation was observed between HCT and Δt, and it was estimated by a third-degree polynomial equation (R^2^ = 0.912). Having considered the range of the Δt and HCT values used for the calibration, which reflect the range of interest for this application, hence 30% to 70% hematocrits, the following model in the range (15s < Δt < 58s) was proposed and reported in Equation (1), The HCT% estimation model identified on the calibration data:

(1) $$\mathrm{HCT}\left(\Delta t\right)\%=0.001\times \Delta t^{3}-0.1473\times \Delta t^{2}+7.218\times \Delta t-49.07$$

**Figure 5:**
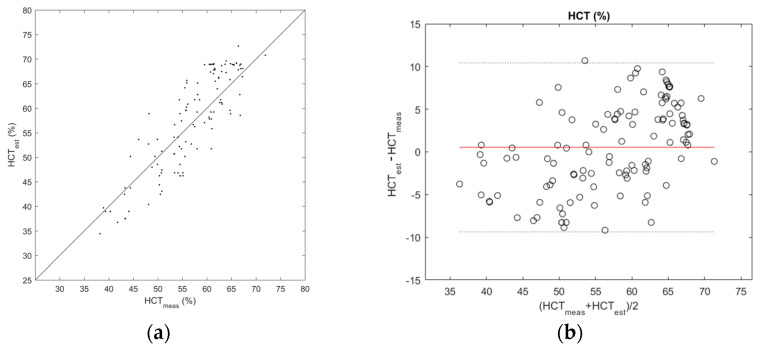
(**a**) Estimated hematocrit vs. measured hematocrit; (**b**) Bland–Altman plot for the estimated hematocrit (HCTest) and measured hematocrit (HCTmeas). The red line indicates the mean difference between the two datasets, and the black dotted lines indicate 95% confidence intervals.

The identified model was subsequently used to estimate HCT values (HCT_est_) on a test set, which were then compared with measured ones (HCT_meas_). In Figure 5a, the estimated hematocrit HCT_est_ values were plotted against HCT_meas_ for each measurement. The results showed dispersion around the identity line, and a significant linear correlation was observed between HCT_est_ and HCT_meas_ (r = 0.87, *p* < 0.001). The mean absolute error was 4.29%, while the maximum absolute error was 10.69%. The comparison of HCT_est_ and HCT_meas_ by Bland–Altman analysis (Figure 5b) showed a good agreement with a low mean difference of 0.53 ± 5.04% and a slight trend of overestimation for higher hematocrit values.

## 4. Discussion

Hematocrit is a crucial physical parameter that provides information about health status and pathological conditions in both adults and newborn patients. While modern countries are equipped with gold standard measurement methods, such as automated laboratory analyzers, in LMICs, there is often a lack under structural, technological, and organizational aspects. For these realities, the need for inexpensive, fast, and portable point-of-care devices is strong [17,22,23]. Taking these requirements as a starting point, the aim of this work was to develop a system capable of meeting them and addressing a newborn’s specific needs.

In this work, we proposed a novel HCT estimation method based on penetration velocity in lateral flow test strips and a light reflectance meter complying with the requirements in LMIC scenarios. The comparison in a clinical environment conducted in the present study confirmed the technical feasibility of this approach and its applicability for the screening of newborns in difficult scenarios, such as those found in LMIC, even though its accuracy has great margins of improvement.

In particular, the test phase using 20% of collected data resulted in a coefficient of determination of 0.912, which may have been limited by the need for a third-grade interpolation function to properly fit input data; the function itself (as reported in Figure 4) features a higher slope in the 30%–60% hematocrit range while being flatter for higher levels reaching saturation for 70% hematocrit; this aspect itself might not prevent the usage of this method in clinical practice if intended as a screening tool for newborn patients, having observed reference ranges defined in previous literature [1]: in the first day of life, the average hematocrit value is about 53%, reducing monotonically in the following 28 days, and the 95% reference range is below 65% in the worst case. The subsequent comparison with a reference method performed in a clinical environment demonstrated the applicability of the proposed method, resulting in a good agreement between estimated and measured HCT values. In spurious cases, the measured value discarded by around 10% or more with respect to the real value provided by the reference method. Observing the distribution of errors in the Bland-Altman plot, its increased density in 65%–70% HCT can be interpreted as a consequence of the reduction in slope of the model characteristic. Furthermore, it is not possible to exclude that some errors derive from abnormal conditions, including singular blood sample features or imprecise sample loading on the test strip.

All the methods taken as reference were capable of effectively measuring HCTs below 35%, with [25] being the best of them, reaching 14.2%. On the other hand, only one of them tested HCTs equal to or higher than those tested by this study [24], a level at which this method presents its better accuracy; three of them tested ranges of HCTs having maximum values of approximately 50%, hence partially covering reference ranges for neonates [15,23,25].

Timewise, it was confirmed that this method allows a fast response and a shorter turnaround time with minimum effort required: not considering that the amount of time required to collect the sample, which is present in any method, results could be retrieved within 3 min. The paper-based method proposed by [18,19,24] presented a longer turnaround time of 30 min, subsequently reduced to 10 min. The image-analysis-based method by [26] was not determined in these terms, but it could be speculated that about 5 min is necessary for sample deposition, sample analysis, and elaboration. The fastest method among those chosen as a reference was the impedance-based device, which, according to authors, is instantaneous [25].

Another point of view is portability: the paper-based device from Berry, S.B. et al. was easily the most portable due to its pure paper design that did not require any energy supply [21]. The proposed method is based on lateral flow test strips and a reflectance meter, which needs a source to power its electronic components. Being a relatively simple device, the reflectance meter is so small that it can be proposed as a handheld device powered by batteries. In this way, portability would be preserved, and the constraints highlighted in pertinent literature are satisfied [22,23]. Devices that incorporate reflectance photometers, such as the device reported in a recent study [30], can be available for LMIC scenarios. The impedance-based device also shows good portability and low energy requirements [25]. All other aforementioned methods did not directly address this concern.

The last comparison is made from an accuracy perspective: the thermometer-style device ensured a good linearity with both plasma and whole blood, being understood that the nonquantitative principle of the system could lead to user errors [22]. This proposed penetration-velocity-based method did not reach such high performance, suggesting the need for future research and improvement of the algorithm underneath.

Overall, with respect to the others, this method operates in an adequate range of hematocrit for neonatal screening, and its turnaround time is shorter than both the majority of compared methods and the most diffused state of the art methods. Moreover, it is deemed to be applicable in LMICs due to its portability, hence its usability both in- and outside clinical facilities. The proposed method showed a significantly lower accuracy than gold standards, and in its current state of development, it is not suitable for diagnostic purposes; nevertheless, it could be useful if intended as a fast, portable, inexpensive, and easy-to-use screening tool. In this sense, clinical infrastructures and facilities that already have such a system would gain a secondary functionality at no further costs, with no hardware changes on the reader or on the test strips. Future research will deepen the knowledge on this method, mostly in the following aspects: (I) enhancement of the algorithm to reach better accuracy; (II) enhancement of optical measurement hardware, such as test strips and LEDs; and (III) reproducibility of the results.

## 5. Conclusions

The penetration-velocity-based method proposed by the present study was characterized and subsequently evaluated by comparison with a reference method. Although not reaching comparable diagnostic performance, it provided sufficient accuracy to be used as a screening system. Moreover, a comparison with other developing methods allowed for validating this system under aspects such as accepted input range, turnaround time, and portability, leading to promising future development.

## Data Availability

Anonymized data are available upon reasonable request to the corresponding author.

## References

[1] Jopling J., Henry E., Wiedmeier S.E., Christensen R.D. (2009). Reference Ranges for Hematocrit and Blood Hemoglobin Concentration during the Neonatal Period: Data from a Multihospital Health Care System. Pediatrics.

[2] Scholkmann F., Ostojic D., Isler H., Bassler D., Wolf M., Karen T. (2019). Reference Ranges for Hemoglobin and Hematocrit Levels in Neonates as a Function of Gestational Age (22–42 Weeks) and Postnatal Age (0–29 Days): Mathematical Modeling. Children.

[3] Bosshart M., Stover J.F., Stocker R., Asmis L.M., Feige J., Neff T.A., Schuepbach R.A., Cottini S.R., Béchir M. (2010). Two different hematocrit detection methods: Different methods, different results?. BMC Res. Notes.

[4] Jedrzejewska-Szczerska M., Gnyba M. (2011). Optical Investigation of Hematocrit Level in Human Blood. Acta Phys. Pol. A.

[5] Colombatti R., Sainati L., Trevisanuto D. (2016). Anemia and transfusion in the neonate. Semin. Fetal Neonatal Med..

[6] Quinn J.G., Tansey E.A., Johnson C.D., Roe S.M., Montgomery L.E.A. (2016). Blood: Tests used to assess the physiological and immunological properties of blood. Adv. Physiol. Educ..

[7] Livshits L., Bilu T., Peretz S., Bogdanova A., Gassmann M., Eitam H., Koren A., Levin C. (2022). Back to the “Gold Standard”: How Precise Is Hematocrit Detection Today? Novel ImageJ-based approach for the precise hematocrit measurement. Mediterr. J. Hematol. Infect. Dis..

[8] Lamola A.A., Bhutani V.K., Wong R.J., Stevenson D.K., McDonagh A.F. (2013). The effect of hematocrit on the efficacy of phototherapy for neonatal jaundice. Pediatr. Res..

[9] Kemper A.R., Newman T.B., Slaughter J.L., Maisels M.J., Watchko J.F., Downs S.M., Grout R.W., Bundy D.G., Stark A.R., Bogen D.L. (2022). Clinical Practice Guideline Revision: Management of Hyperbilirubinemia in the Newborn Infant 35 or More Weeks of Gestation. Pediatrics.

[10] Gebretsadkan G., Ambachew H. (2015). The Comparison between Microhematocrit and Automated Methods for Hematocrit Determination. Int. J. Blood Res. Disord..

[11] Mondal H., Lotfollahzadeh S. (2022). Hematocrit. StatPearls.

[12] Verbrugge S.E., Huisman A. (2015). Verification and Standardization of Blood Cell Counters for Routine Clinical Laboratory Tests. Clin. Lab. Med..

[13] Buttarello M. (2016). Laboratory diagnosis of anemia: Are the old and new red cell parameters useful in classification and treatment, how?. Int. J. Lab. Hematol..

[14] Gavala A., Myrianthefs P. (2017). Comparison of point-of-care versus central laboratory measurement of hematocrit, hemoglobin, and electrolyte concentrations. Heart Lung.

[15] Woldie M., Feyissa G.T., Admasu B., Hassen K., Mitchell K., Mayhew S., McKee M., Balabanova D. (2018). Community health volunteers could help improve access to and use of essential health services by communities in LMICs: An umbrella review. Health Policy Plan..

[16] Lehmann U., Dieleman M., Martineau T. (2008). Staffing remote rural areas in middle- and low-income countries: A literature review of attraction and retention. BMC Health Serv. Res..

[17] Caldwell A., Young A., Gomez-Marquez J., Olson K.R. (2011). Global Health Technology 2.0. IEEE Pulse.

[18] Li X., Cui K., Xiu M., Zhou C., Li L., Zhang J., Hao S., Zhang L., Ge S., Huang Y. (2022). In situ growth of WO_3_/BiVO_4_ nanoflowers onto cellulose fibers to construct photoelectrochemical/colorimetric lab-on-paper devices for the ultrasensitive detection of AFP. J. Mater. Chem. B.

[19] Zhou C., Cui K., Liu Y., Li L., Zhang L., Hao S., Ge S., Yu J. (2021). Bi_2_S_3_@MoS_2_ Nanoflowers on Cellulose Fibers Combined with Octahedral CeO_2_ for Dual-Mode Microfluidic Paper-Based MiRNA-141 Sensors. ACS Appl. Mater. Interfaces.

[20] Waller A.W., Toc M., Rigsby D.J., Gaytán-Martínez M., Andrade J.E. (2019). Development of a Paper-Based Sensor Compatible with a Mobile Phone for the Detection of Common Iron Formulas Used in Fortified Foods within Resource-Limited Settings. Nutrients.

[21] Bender A.T., Sullivan B.P., Zhang J.Y., Juergens D.C., Lillis L., Boyle D.S., Posner J.D. (2021). HIV detection from human serum with paper-based isotachophoretic RNA extraction and reverse transcription recombinase polymerase amplification. Analyst.

[22] Smith S., Land K., Joubert T.-H. Emerging Technology Solutions Towards REASSURED Point-of-Need Diagnostics. Proceedings of the 2021 IEEE 21st International Conference on Nanotechnology (NANO).

[23] Murray L.P., Mace C.R. (2020). Usability as a guiding principle for the design of paper-based, point-of-care devices—A review. Anal. Chim. Acta.

[24] Berry S.B., Fernandes S.C., Rajaratnam A., DeChiara N.S., Mace C.R. (2016). Measurement of the hematocrit using paper-based microfluidic devices. Lab Chip.

[25] Fernandes S.C., Baillargeon K.R., Mace C.R. (2019). Reduction of blood volume required to perform paper-based hematocrit assays guided by device design. Anal. Methods.

[26] Ben F.D., Biasizzo J., Curcio F. (2019). A fast, nondestructive, low-cost method for the determination of hematocrit of dried blood spots using image analysis. Clin. Chem. Lab. Med. (CCLM).

[27] Miller J.H. (2013). An On-card Approach for Assessment of Hematocrit on Dried Blood Spots which Allows for Correction of Sample Volume. J. Anal. Bioanal. Tech..

[28] Punter-Villagrasa J., Cid J., Páez-Avilés C., Rodríguez-Villarreal I., Juanola-Feliu E., Colomer-Farrarons J., Miribel-Català P.L. (2015). An Instantaneous Low-Cost Point-of-Care Anemia Detection Device. Sensors.

[29] Cohen E., Kramer M., Shochat T., Goldberg E., Krause I. (2017). Relationship between hematocrit levels and intraocular pressure in men and women: A population-based cross-sectional study. Medicine.

[30] Greco C., Iskander I.F., Houchi S.Z.E., Rohsiswatmo R., Rundjan L., Ogala W.N., Ofakunrin A.O.D., Moccia L., Hoi N.T.X., Bedogni G. (2018). Diagnostic Performance Analysis of the Point-of-Care Bilistick System in Identifying Severe Neonatal Hyperbilirubinemia by a Multi-Country Approach. eClinicalMedicine.

